# Lifetime of ground conformational state determines the activity of structured RNA

**DOI:** 10.21203/rs.3.rs-2879957/v1

**Published:** 2023-05-26

**Authors:** Rhese D. Thompson, Derek L. Carbaugh, Joshua R. Nielsen, Ciara M. Witt, Rita M. Meganck, Atul Rangadurai, Bo Zhao, Jeffrey P. Bonin, Nathan I. Nicely, William F. Marzluff, Aaron T. Frank, Helen M. Lazear, Qi Zhang

**Affiliations:** 1 Department of Biochemistry and Biophysics, University of North Carolina at Chapel Hill, Chapel Hill, NC, USA; 2 Department of Microbiology and Immunology, University of North Carolina at Chapel Hill, Chapel Hill, NC, USA; 3 Department of Biophysics and Chemistry, University of Michigan, Ann Arbor, MI, USA; 4 Department of Chemistry, University of North Carolina at Chapel Hill, Chapel Hill, NC, USA; 5 Department of Pharmacology, University of North Carolina at Chapel Hill, Chapel Hill, NC, USA; 6 Department of Biochemistry, Duke University, Durham, NC, USA; 7 RNA Discovery Center, University of North Carolina at Chapel Hill, Chapel Hill, NC, USA

## Abstract

Biomolecules continually sample alternative conformations. Consequently, even the most energetically favored ground conformational state has a finite lifetime. Here, we show that, in addition to the 3D structure, the lifetime of a ground conformational state determines its biological activity. Using hydrogen-deuterium exchange nuclear magnetic resonance spectroscopy, we found that Zika virus exoribonuclease-resistant RNA (xrRNA) encodes a ground conformational state with a lifetime that is ~10^5^–10^7^ longer than that of canonical base pairs. Mutations that shorten the apparent lifetime of the ground state without affecting its 3D structure decreased exoribonuclease resistance *in vitro* and impaired virus replication in cells. Additionally, we observed this exceptionally long-lived ground state in xrRNAs from diverse infectious mosquito-borne flaviviruses. These results demonstrate the biological significance of the lifetime of a preorganized ground state and further suggest that elucidating the lifetimes of dominant 3D structures of biomolecules may be crucial for understanding their behaviors and functions.

Biomolecules fold into dominant ground-state conformations that dynamically equilibrate with alternative short-lived and low-abundance excited conformational states^1–3^. A growing number of studies have shown that the lifetime or abundance of the excited conformational states can be a crucial determinant for the activities of protein^4–8^, DNA^9,10^, and RNA^11–13^. Because biomolecules continually sample alternative conformations along the free energy landscape, even the energetically most favored and most populated ground state has a finite lifetime, which may contribute to biology activities. However, studies examining the functional significances of ground conformational states have almost exclusively focused on their three-dimensional (3D) structures and thermodynamic stabilities, which are increasingly accessible for diverse biomolecules benefiting from advances in cryo-EM and structure predictions^14,15^. Here, we report the discovery that the ground conformational state formed by the exoribonuclease-resistant RNAs (xrRNAs) from mosquito-borne flaviviruses (MBFVs) has a conserved and exceptionally long lifetime. This prolonged lifetime enables the viral RNA to create a steady molecular wall that lasts long enough to resist host exonuclease degradation of the viral genome, thus promoting viral replication.

xrRNAs are an emerging class of non-coding RNAs that adopt unique tertiary structures and endure tremendous unidirectional mechanical stress^16,17^. In MBFVs, which are important endemic human pathogens including Zika, dengue, and West Nile viruses that cause hemorrhagic fever, encephalitis, and congenital disabilities^18^, xrRNAs are located in the 3’ untranslated regions of these positive-sense single-stranded RNA viruses^19,20^, stalling the host 5’-3’ exonuclease Xrn1 and producing subgenomic flavivirus RNAs (sfRNAs) that antagonize host antiviral responses and promote viral replication^21,22^. The high-resolution crystal structure of xrRNA from Zika virus (ZIKV) has revealed a core conformation at its 5’-end encapsulated by a long-range pseudoknot^23^. While the concurrence of both the core and the pseudoknot motifs has been shown to be critical for maintaining high Xrn1-resistance, the molecular mechanism via which xrRNAs resist exoribonuclease remains elusive, especially given that the core structure is preserved even in the absence of pseudoknot as shown in the crystal structure of xrRNA from Murray Valley encephalitis virus (MVEV)^24^.

In our study, we used *in vitro* Xrn1 digestion assay and *in cell* viral replication assay to examine how mutations perturbing the pseudoknot content of ZIKV xrRNA impact its Xrn1 resistance *in vitro* and ZIKV replication in mammalian Vero cells. Strikingly, we found that mutations significantly decreased Xrn1 resistance and impaired ZIKV replication in mammalian Vero cells even though the pseudoknot variants not only folded into essentially identical 3D crystal structures as wild-type ZIKV xrRNA but also had similar thermal stabilities. Using hydrogen-deuterium exchange (HDX) nuclear magnetic resonance (NMR) spectroscopy, we uncovered a ‘hidden’ conformational kinetics embedded in wild-type ZIKV xrRNA that evaded detection by conventional structural biology techniques. We found that a conserved G–C–C triple, which provides a structural anchor on the ring-like architecture of ZIKV xrRNA, encodes an extraordinary slow base-opening dynamics with a lifetime of 274 ± 14 min at 17°C that is ~10^5^-10^7^ longer than those of canonical base pairs. In contrast, functionally weakened pseudoknot variants of ZIKV xrRNA allosterically reduced the apparent lifetime of this pivotal conformational state without altering the base-triple interaction. Furthermore, we demonstrated that this exceptionally long-lived ground conformational state of ZIKV xrRNA is robustly observed in xrRNAs from other infectious mosquito-borne flaviviruses, including dengue virus serotype-1, Japanese encephalitis virus, and Saint Louis encephalitis virus. Together, these findings unveil the importance of the lifetime of a ground conformational state in a biological process and further suggest that long-lived base pairs may serve as unique structural motifs in RNA with potentially broad functional roles.

## RESULTS

### Long-range pseudoknot interaction regulates ZIKV xrRNA1 function

**Fig. 1 |:**
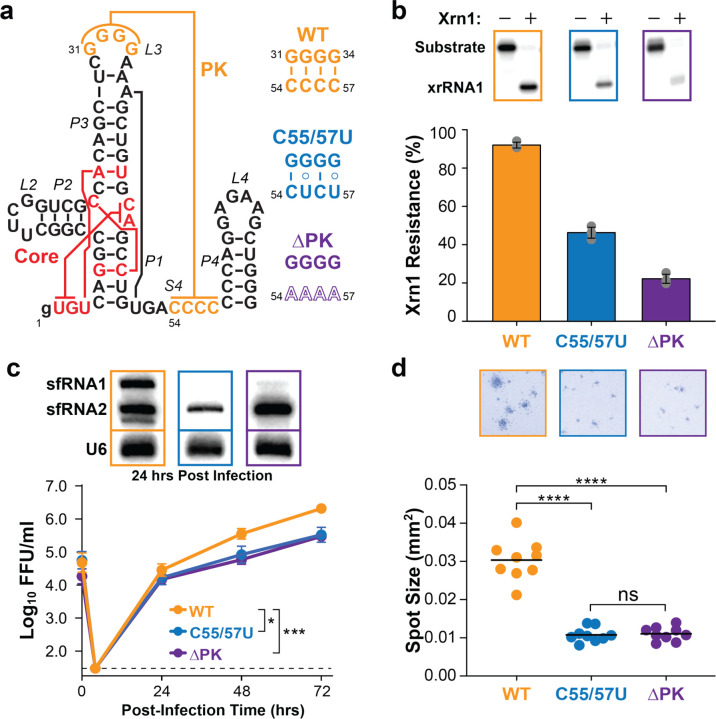
Pseudoknot interaction modulates Xrn1 resistance of Zika virus xrRNA1. **a,** Secondary structure of ZIKV xrRNA1 and pseudoknot sequences of wild-type (WT), weakening (C55/57U), and pseudoknot-less (DPK) mutants. **b,** *In vitro* Xrn1 digestion assays of WT, C55/57U, and DPK ZIKV xrRNA1s with ^32^P body labeling. Plotted are average percentages of Xrn1 resistance from three replicates with standard deviations as error bars. **c,** Quantification of viral replication of WT and mutant ZIKV in Vero cells. Viral titers were quantified using focus-forming assays and reported in the unit of focusforming units per milliliter (FFU/ml). Plotted are average values from 15–18 replicates from 5–6 independent experiments with standard error of the mean as error bars, where * and *** denote p-values <0.05 and <0.001, respectively, for statistical differences between WT and mutant ZIKV. Northern blots of sfRNAs from ZIKV-infected Vero cells are shown together with U6 RNA as an internal control. **d,** Quantification of cell-to-cell spread of WT and mutant ZIKV in Vero cells. Plotted are mean areas of infectious foci from 9 wells of 1 representative plate, where ns and **** denote p-values >0.05 and <0.0001, respectively, for statistical differences between WT and mutant ZIKV.

We examined the functional consequences of introducing mutations to the pseudoknot of xrRNA while keeping the core residues intact. For these studies, we designed two constructs based on the 5’-upstream xrRNA of the two xrRNAs in ZIKV (Fig. 1a), which has been widely employed for studying the biochemical, biophysical, and mechanical properties of xrRNAs^23,25–30^. In the first mutant, xrRNA1^C55/57U^, which aims to weaken the pseudoknot, we changed the second and fourth G–C base pairs in the long-range pseudoknot to G–U wobbles. In the second mutant, xrRNA1^ΔPK^, we replaced all four cytosines in the S4 region with adenines to eliminate the pseudoknot interaction.

We first characterized how pseudoknot variations affect *in vitro* activities of xrRNA. Consistent with previous studies, the wild-type xrRNA1^WT^ showed strong Xrn1resistance of 92% ± 2% in the *in vitro* Xrn1 digestion assay. While the xrRNA1^DPK^ mutant had a substantially reduced resistance of 22% ± 2%, the xrRNA1^C55/57U^ mutant exhibited an intermediate resistance of 46% ± 3% between the wild type and the pseudoknot-less mutant (Fig. 1b), suggesting that the context of the long-range pseudoknot interaction influences the *in vitro* activity of xrRNAs.

Next, we evaluated the cellular effects of pseudoknot variants on viral replication. We introduced xrRNA1^C55/57U^ and xrRNA1^DPK^ mutations into the ZIKV genome (Extended Data Fig. 1a,b) and subsequently measured viral replication in mammalian Vero cells. As can be seen, both mutations significantly impaired ZIKV replication compared to the WT virus, resulting in 0.626 Log_10_ and 0.780 Log_10_ reduction at 48 hours post-infection for xrRNA1^C55/57U^ and xrRNA1^DPK^, respectively (Fig. 1c). Northern blots of infected cells further supported that these replication defects were due to defects in xrRNA-dependent sfRNA accumulation (Fig. 1c). In cells infected with WT ZIKV, two sfRNAs (sfRNA1 and sfRNA2) were evident, corresponding to viral genomic RNAs digested by Xrn1 to xrRNA1 and xrRNA2 sites, respectively. Interestingly, despite the near complete Xrn1-resistance of xrRNA1 *in vitro*, the similar accumulation of sfRNA1 and sfRNA2 in infected cells suggests that xrRNA effectiveness can be influenced by the infected cell environment, resulting in incomplete Xrn1 resistance. In contrast, sfRNA1 was largely undetectable in xrRNA1^C55/57U^- and xrRNA1^DPK^-infected cells (Fig. 1c). Furthermore, xrRNA1^C55/57U^ and xrRNA1^DPK^ mutants exhibited impaired cell-to-cell spread, producing significantly smaller infectious foci (spot size^C55/57U^ = 1.08 ± 0.06 × 10^−2^ mm^2^ and spot size^ΔPK^ = 1.10 ± 0.06 × 10^−2^ mm^2^) compared to the WT virus (spot size^WT^ = 3.04 ± 0.18 × 10^−2^ mm^2^) (Fig. 1d and Extended Data Fig. 1c). Together, these results suggest that the integrity of the pseudoknot interaction in xrRNA is crucial for *in vitro* Xrn1 resistance and ZIKV replication and spread in cell culture.

### Structure of ZIKV xrRNA1 mutant with reduced Xrn1 resistance

**Fig. 2 |:**
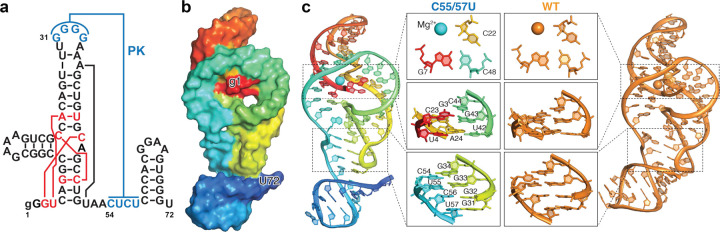
Structure of ZIKV xrRNA1s. **a,** Secondary structure of the ZIKV xrRNA1 C55/57U construct in crystallography study. **b,** Electron density map, 2mFo-DFc, shown at 1σ on ZIKV xrRNA1 C55/57U construct, and surface representation of the crystal structure that highlights the ring architecture. **c,** Structural comparison between C55/57U (rainbow colored) and WT (orange, PDB: 5TPY) ZIKV xrRNA1s.

To obtain structural insights into the observed pseudoknot-dependent xrRNA activity, we solved a crystal structure of the pseudoknot-weakening xrRNA1^C55/57U^ mutant at 3.15 Å resolution (Fig. 2a,b, Extended Data Fig. 2, and Supplementary Table 1), which complements the existing structures of pseudoknot-less and wild-type xrRNAs from MVEV xrRNA2 and ZIKV xrRNA1, respectively^23,24^. Remarkably, the xrRNA1^C55/57U^ structure is essentially identical to the previously reported crystal structure of the WT ZIKV xrRNA1^23^, except for a minor twisting of the P4 stem due to the presence of an additional residue (U72) at the 3’-end in our construct (Fig. 2a). The ring-like architecture formed from A37 to A52, which encapsulates the 5’-end of the xrRNA that directly encounters Xrn1, is readily visible in the xrRNA1^C55/57U^ structure (Fig. 2b). The core interactions in the WT ZIKV xrRNA1 are all maintained in the C55/57U mutant, including G7–C48–C22 and A24–U42–U4 base-triples, and C23–G43 and G3–C44 base pairs (Fig. 2c). The long-range pseudoknot is also well-formed with alternating G–C and G–U base pairs in the xrRNA1^C55/57U^ structure (Fig. 2c).

To characterize the conformational behavior of the xrRNA1^C55/57U^ mutant under solution conditions, we then applied NMR on shorter xrRNA constructs lacking the P4 stem (Extended Data Fig. 3a), shown previously to be dispensable for Xrn1 resistance^31^. As can be seen, the presence of the G–U wobble in the xrRNA1^C55/57U^ mutant is evidenced by the unique G–U cross-peaks in the NMR ^1^H-^1^H nuclear Overhauser effect spectroscopy (NOESY) spectrum of the C55/57U mutant but not the wild-type xrRNA1 (Extended Data Fig. 3a). Thus, the NMR data indicate that the xrRNA1^C55/57U^ mutant forms a conformation with an intact pseudoknot, as shown in its crystal structure (Fig. 2c).

To gain insights into the effects of pseudoknot variants on the overall stability of xrRNAs, we conducted additional UV melting experiments on these NMR constructs. Surprisingly, all three constructs – the wild-type, the pseudoknot-weakening, and the pseudoknot-less xrRNA1s – showed nearly identical melting temperatures, with *T*_m_^WT^ = 75.8 ± 0.1 °C, *T*_m_^C55/57U^ = 75.6 ± 0.4 °C, and *T*_m_^ΔPK^ = 77.5 ± 0.3 °C (Extended Data Fig. 3b). These results suggest that factors other than the pseudoknot, such as the helical regions of xrRNA, are likely the determinants of the global thermal melting profile, resulting in undetectable changes in global xrRNA stability due to pseudoknot mutations.

### ZIKV xrRNA1 encodes a long-lived ground conformational state

**Fig. 3 |:**
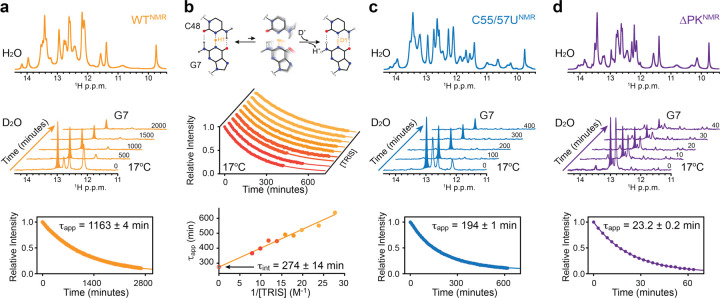
Conformational dynamics of ZIKV xrRNA1s by HDX NMR. **a,** HDX NMR of WT xrRNA1 NMR construct at 17°C. Shown are ^1^H NMR spectra of the imino proton region in H_2_O and D_2_O. Time-dependent peak intensities of G7 imino proton upon dissolving in D_2_O are fit to a mono-exponential decay. Reported is the average apparent lifetime with s.d. estimated from fitting *n* = 241 data points. **b,** Tris-dependent HDX NMR of WT xrRNA1 NMR construct at 17°C. Shown is a schematic diagram that depicts the H-D exchange process of the G7 imino proton. Time-dependent peak intensities of G7 imino proton upon dissolving in D_2_O are plotted as a function of Tris concentration. Individual average apparent lifetimes with s.d. estimated from *n* = 49 to 100 data points are fit linearly to 1/[Tris] to extract the intrinsic lifetime of the G7 imino proton. **c-d,** HDX NMR of C55/57U (**c**) and DPK (**d**) xrRNA1 NMR constructs at 17°C. Shown are ^1^H NMR spectra of the imino proton region in H_2_O and D_2_O. Time-dependent peak intensities of G7 imino proton upon dissolving in D_2_O are fit to a mono-exponential decay. Reported are average apparent lifetimes with s.d. estimated from fitting *n* = 215 and 23 data points for C55/57U and DPK, respectively.

How do these pseudoknot variants of xrRNA1 with similar structures and global thermal stabilities exhibit different exonuclease resistances? Xrn1 digestion requires opening structured base pairs to generate single-stranded RNA that enters the active site of Xrn1. Hence, differences in the local unfolding propensities of crucial structural elements could determine the different behavior of these pseudoknot mutants. To test this possibility, we used hydrogen-deuterium exchange (HDX) NMR, a powerful approach for probing the base-pair opening dynamics in nucleic acids^32–36^. In a time resolved HDX experiment, an RNA sample is dissolved in D_2_O, and the exchange of G and U imino protons with deuterons is observed as a function of time. Typically, these imino protons exchange with deuterons within milliseconds. Remarkably, upon being resuspended in D_2_O for several hours, distinct imino proton signals in the xrRNA1^WT^ NMR construct remained in the ^1^H NMR spectra (Fig. 3a). In particular, the imino proton signal of G7 (Extended Data Fig. 4a), universally conserved in MBFV xrRNAs, persisted for over 24 hours at 17°C with an apparent lifetime (τ_app_) of 1163 ± 4 min (Fig. 3a).

To examine whether the long lifetime of the G7 imino proton was due to a slow rate of base-pair opening, we carried out HDX NMR measurements as a function of Tris concentration, where the base form of Tris serves as an HDX catalyst for probing base opening dynamics^37^. Linear extrapolation of the apparent proton lifetimes to infinite base concentration allowed us to determine the intrinsic lifetime of the G7–C48 base pair, τ_int_ = 274 ± 14 min at 17°C, hence a rate of base pair opening of 6.1 ± 0.3 × 10^−5^ s^−1^ (Fig. 3b). This intrinsic lifetime of G7 in xrRNA1^WT^ is ~10^5^-10^7^-fold longer than those of canonical Watson-Crick base pairs, typically in tens of milliseconds^38–41^. Using this distinct kinetic signature, NMR HDX profiles revealed striking perturbations in conformational dynamics for the functionally impaired xrRNA1 mutants, in which the apparent lifetime of G7 imino proton exhibited a 6-fold and 50-fold decrease from τ_app_ = 1163 ± 4 min in xrRNA1^WT^ to τ_app_ = 194 ± 1 min in xrRNA1^C55/57U^ and 23.2 ± 0.2 min in xrRNA1^ΔPK^ at 17°C (Fig. 3c,d and Extended Data Fig. 4b,c).

Structurally, G7 forms the G7–C48–C22 base triple and serves as an anchoring residue on the ring-like architecture of xrRNA, which encapsulates the 5’-end of the RNA from Xrn1 digestion. Hence, the observed differences in the apparent lifetime of G7 among pseudoknot variants may reflect changes in the overall plasticity of the ring-like architecture, which can impair the mechanical strength of xrRNA without significantly perturbing its ground-state conformation. As it has been shown that xrRNA can halt Xrn1 for a substantial amount of time before disassociation^42^, these potential mechanical breaches in xrRNA architecture, when coupled with the irreversible nature of degradation, could be exploited by Xrn1 and give rise to transient breakthroughs in its resistance.

### The ground-state lifetime of MBFV xrRNA directs the exonuclease resistance

**Fig. 4 |:**
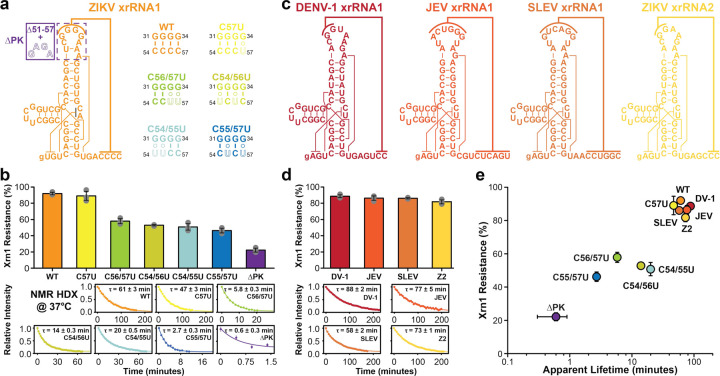
Conformational dynamics and Xrn1 resistance of MBFV xrRNAs. **a,** Secondary structures of WT and mutant ZIKV xrRNA1s. **b,** Dynamic and functional analyses of WT and mutant ZIKV xrRNA1s at 37°C. Shown are the secondary structure of ZIKV xrRNA1 and pseudoknot sequences of WT and mutants. Plotted are average percentages of Xrn1 resistance from three replicates of *in vitro* Xrn1 digestion assay with standard deviations as error bars. Time-dependent peak intensities of G7 imino proton upon dissolving in D_2_O are plotted for each construct and fit to mono-exponential decays. Reported are average apparent lifetimes with s.d. estimated from fitting two independent measurements with a total of *n* = 59, 40, 27, 92, 94, 27, 4 data points for WT, C57U, C56/57U, C54/56U, C54/55U, C55/57U, and DPK, respectively. **c,** Secondary structures of other mosquito-borne flavivirus xrRNAs. **d,** Dynamic and functional analyses of other mosquito-borne flavivirus xrRNAs at 37°C. Plotted are average Xrn1 resistances of dengue virus 1 xrRNA1 (DENV-1), Japanese encephalitis virus xrRNA1 (JEV), Saint Louis encephalitis virus xrRNA1 (SLEV), and ZIKV xrRNA2 (Z2) from three replicates of *in vitro* Xrn1 digestion assay with standard deviations as error bars. Time-dependent peak intensities of G7 imino proton upon dissolving in D_2_O are plotted for each construct and fit to mono-exponential decays. Reported are average apparent lifetimes with s.d. estimated from fitting two independent measurements with a total of *n* = 82, 66, 65, 74 data points for DENV-1, JEV, SLEV, and Z2, respectively. **e,** Diagram of Xrn1 resistances and the apparent lifetimes of G7 imino proton of xrRNAs.

To investigate the functional significance of the lifetime of the ground conformational state more comprehensively, we initially extended our three ZIKV xrRNA1 constructs by including four alternative pseudoknot variants (xrRNA1^C57U^, xrRNA1^C56/57U^, xrRNA1^C54/56U^, and xrRNA1^C54/55U^) with the aim of modifying exonuclease activities (Fig. 4a). NMR characterizations confirmed that the ground conformational states of these new sequences are well-folded, as evidenced by the ^1^H-^1^H NOESY spectra and the detection of signature G7 imino proton resonances (Extended Data Fig. 5). We then performed the *in vitro* Xrn1 digestion assays, and indeed, these pseudoknot variants exhibited varying decreases in activities compared to the wild-type xrRNA1 (Fig. 4b and Extended Data Fig. 5). To closely relate to the biological outcomes, we repeated the HDX NMR experiments on all seven ZIKV xrRNA1 constructs at 37°C, the temperature used for the *in vitro* Xrn1 assays, and observed the apparent lifetime of the G7 imino proton ranging from τ_app_ = 61 ± 3 min in xrRNA1^WT^ to τ_app_ = 0.6 ± 0.3 min in xrRNA1^ΔPK^ (Fig. 4b and Extended Data Fig. 5).

Next, we broadened our characterizations to include the 3’-downstream xrRNA of the two ZIKV xrRNAs (Z2) as well as wild-type xrRNAs from other infectious MBFVs, such as xrRNAs from dengue virus serotype-1 (DV-1), Japanese encephalitis virus (JEV), and Saint Louis encephalitis virus (SLEV) (Fig. 4c). Notably, despite distinct sequences and pseudoknot interactions, these wild-type MBFV xrRNAs displayed similar signature NMR signals of a long-lived G7 imino proton, as observed in ZIKV xrRNA1 (Extended Data Fig. 6). These xrRNAs not only demonstrated robust *in vitro* Xrn1 resistances from 82% ± 2% to 89% ± 2%, but their corresponding G7s also exhibited long apparent lifetimes ranging from τ_app_ = 58 ± 2 min in SLEV to τ_app_ = 88 ± 2 min in DV-1 at 37°C (Fig. 4d and Extended Data Fig. 6). Collectively, the results from these eleven xrRNA constructs reveal that the lifetime of the ground conformational state directly influences the exonuclease resistance; the longer the ground-state lifetime, the greater the inhibitory efficacy (Fig. 4e). Furthermore, the findings observed among all wild-type xrRNAs suggest that MBFVs have evolved to maintain prolonged lifetimes for their preorganized xrRNA ground conformational states, providing the necessary structural and kinetic resilience to withstand Xrn1 digestion.

### Constant-force simulation of xrRNA exonuclease resistance

**Fig. 5 |:**
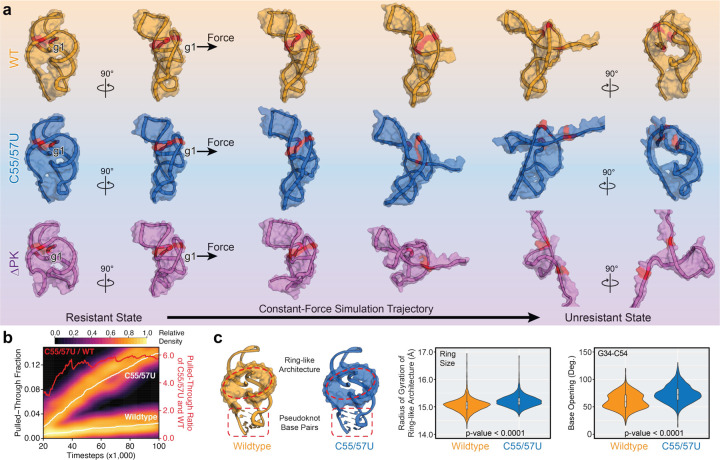
Xrn1 resistance of ZIKV xrRNA1s via constant-force simulations. **a,** Representative trajectories of WT, C55/57U, and DPK ZIKV xrRNA1s from constantforce molecular dynamics (CFMD) simulations at 400 pN. Shown are states of xrRNA1s captured at the same timesteps along trajectories. The conserved G7–C48–C22 triple is highlighted in red. **b,** Accumulated pulled-through fractions of WT and C55/57U xrRNA1s as a function of timesteps in CFMD simulations. The schematic diagram depicts a pulled-through event as a disrupted G7–C48–C22 triple. **c,** Distribution of the sizes of the ring architecture of WT and C55/57U xrRNA1s in CFMD simulations. Plotted are receiver operating characteristic (ROC) analyses of the ring size and pulledthrough event from WT (orange), C55/57U (blue), and combined (black) trajectories with corresponding area under the curve (AUC) values. Distribution of the opening degrees of the pseudoknot base pair G34–C54 of WT and C55/57U xrRNA1s in CFMD simulations. Plotted are ROC analyses of the base opening and the ring architecture opening from WT (orange), C55/57U (blue), and combined (black) trajectories with corresponding AUC values.

To explore potential molecular mechanisms for the observed pseudoknot-dependent exonuclease resistance, we carried out constant-force molecular dynamics (CFMD) simulations^43,44^ on the wild-type (xrRNA1^WT^), pseudoknot-weakened (xrRNA1^C55/57U^), and pseudoknot-less (xrRNA1^C55/57U^) xrRNAs, for which high-resolution crystal structures are available. In these simulations, a constant pulling force is applied on the 5’-end of the RNA, mimicking the Xrn1 degradation process. Figure 5a shows representative CFMD trajectories of xrRNA1^WT^, xrRNA1^C55/57U^, and xrRNA1^ΔPK^ at a 400-pN pulling force (Supplementary Movies 1 and 2). Without the pseudoknot interactions, xrRNA1^ΔPK^ becomes globally unstructured upon pulling with disrupted G7–C48–C22 base triple. In comparison, both xrRNA1^WT^ and xrRNA1^C55/57U^ preserve their ring-like structures with fewer pseudoknot interactions yet still allow sufficient space for pulling through the 5’-end residues, which is consistent with a recent simulation work on the wild-type ZIKV xrRNA1^28^. Interestingly, despite overall similarities, the xrRNA1^C55/57U^ trajectories exhibited higher frequencies of ‘pulled-throughs,’ defined as a disassociation of G7 from the base triple, compared to those of the wild-type xrRNA1.

We then conducted CFMD simulations across a broad range of pulling forces to benchmark proper force strengths for characterizing structural features associated with ‘pulled-through’ events (Extended Data Fig. 7a). CFMD trajectories from 380–420 pN exhibited a ratio of 5.9 ± 1.4 between xrRNA1^C55/57U^ and xrRNA1^WT^ for simulated ‘pulled-throughs’ (Fig. 5b), which agrees well with the ratio of 6.7 ± 0.4 in their corresponding breakthroughs in resistance measured from *in vitro* Xrn1 digestion assays (Fig. 1b). These CFMD trajectories revealed distinct propensities of the ring-like architecture between xrRNA1^C55/57U^ and xrRNA1^WT^. Despite having nearly identical crystal structures, the xrRNA1^C55/57U^ trajectories consistently displayed a larger ring-like architecture during the pulling process than xrRNA1^WT^ (Fig. 5c). Receiver operating characteristic (ROC) analysis further indicated that the ring size could predict ‘pulled-through’ events with a combined area under the curve (AUC) value of 0.80 (Extended Data Fig. 7b). Structural analysis of these trajectories suggested that the larger ring sizes observed in xrRNA1^C55/57U^ trajectories are likely due to more pronounced opening and stretching of its pseudoknot capping and stem base pairs (Fig. 5c and Extended Data Fig. 7c,d). Together, CFMD simulations imply that the pseudoknot interactions may modulate tightness, hence, structural plasticity of the ring-like architecture of xrRNA for inhibiting Xrn1 digestion, providing a testable model for future experimental investigations.

## DISCUSSION

**Fig. 6 |:**
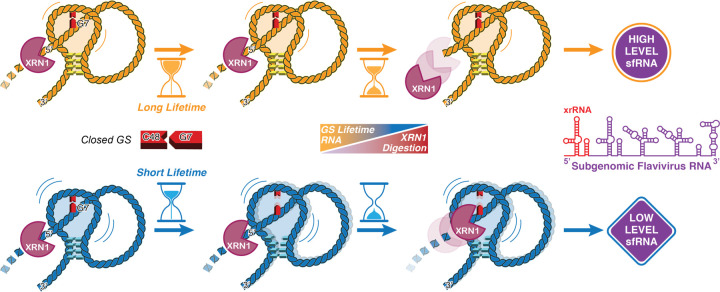
Model for xrRNA-dependent Xrn1 resistance. xrRNA acts as a molecular wall to inhibit Xrn1 digestion towards generating subgenomic flavivirus RNAs to promote viral replication. The strength of exonuclease resistance is influenced by the lifetime of the ground conformational state (GS) of the closed base pair anchored on the ring architecture, which is modulated through the long-range pseudoknot interaction.

In this study, we have uncovered a distinct molecular mechanism for MBFV xrRNAs, where the conformational lifetimes of these vital viral RNAs, which are not evident from static structures, determine their biological function. Exemplified using ZIKV xrRNA1, we found that the two key structural elements of an xrRNA – the core and the pseudoknot –work synergistically not only to create a sophisticated structure to encapsulate the 5’-end of the molecule but also, more importantly, to empower this conformation with ultra-slow base pair opening kinetics and an exceptionally long lifetime to resist Xrn1 digestion. Topologically, xrRNA folds into an L1 pierced lasso, a class of entangled structural motifs that also emerged recently in proteins^45,46^, in which the 5’-end of xrRNA pierces the loop formed through pseudoknot base pairing (Fig. 6). The pseudoknot interactions constrain the overall plasticity of the loop, hence modulating the stability of the piercing strand. For wild-type ZIKV xrRNA1, a strong pseudoknot gives rise to a tight loop and substantially reduces the opening dynamics of the anchoring residue of the piercing strand. Consequently, the ground state (GS) of this base-paired ‘closed’ conformation is kinetically trapped with a prolonged lifetime, resulting in a steady molecular wall that enables high efficacy in Xrn1 inhibition. In contrast, a weakened pseudoknot loosens the loop constraint, shortens the lifetime of the base-paired anchoring residue, and enhances access to the unpaired ‘open’ conformation of the anchor, which leads to an Xrn1-vulnerable piercing strand for digestion.

The distinct kinetic propensity of ZIKV xrRNA1, characterized by an exceptionally long lifetime, is robustly observed in xrRNAs from other infectious mosquito-borne flaviviruses, suggesting that MBFV xrRNAs have evolved to encode not only structures but also conformational kinetics for function. Developing a kinetic model to quantitively depict the relationship between the ground-state lifetime of xrRNA and exonuclease resistance presents a compelling direction for future research. Given that xrRNAs have emerged as a new class of non-coding RNAs^16,17^, we speculate that this kinetics-based mechanism may underlie the function of xrRNAs in species beyond MBFVs. Moreover, the conserved lifetime of the key interaction in MBFV xrRNAs could offer a readily accessible metric for evaluating their activities, with potential implications in developing therapeutics or attenuated vaccines to treat and prevent flavivirus infections.

As fundamental building blocks of nucleic acid structures, base pairs are commonly perceived to open and close rapidly – on the timescale of tens of milliseconds^38–41^ – to accommodate the conformational transitions of RNA and DNA in diverse biological processes^2,35^. Short base-pair lifetimes are essential for numerous biological processes that involve unzipping nucleic acid structures, such as replication, transcription, translation, and protein recognition. However, the ultra-slow base-pair opening dynamics observed in MBFV xrRNAs are associated with their distinct properties of enduring extraordinary mechanical stress. These findings represent one of the first functionally annotated long-lived RNA states. Notably, early applications of HDX NMR since the 1980s have identified base pairs in a few structural motifs with minuteto-hour-long lifetimes in tRNAs^32,36^ and RNA and DNA G-quadruplexes^33,34^. In tRNAs, most of these kinetically trapped base pairs were found in the D arm, a stem region that is involved in maintaining tRNA structural stability. Disease-related mutations in the D arm of mitochondrial tRNAs cause tRNA misfolding and reduce tRNA levels^47^, whereas D-arm-associated mutations that increase tRNA flexibility and facilitate tRNA distortion lead to elevated levels of codon miscoding^48,49^. Therefore, the lifetime of the D arm could play a role in fine-tuning the plasticity of tRNA essential for its function in the context of the ribosome. Moreover, the lifetimes of RNA and DNA G-quadruplexes could also be functionally significant for their mechanical properties, which can stall polymerases and ribosomes in replication, transcription, and translation^50^. With ongoing discoveries of diverse non-coding RNAs, a renaissance of HDX NMR applications on other highly structured RNAs may facilitate the unveiling of long-lived base pairs as unique structural motifs with potentially broad roles in biology.

These findings shed light on the functional role of the lifetime of a ground conformational state in a biochemical process. It is becoming increasingly evident that biomolecules dynamically transition between alternative conformations, each possessing unique structural and kinetic properties for function^1–3^. Our work not only adds the dimension of time to understanding a ground-state structure for function but also poses a new challenge in the field: determining or predicting the lifetime of a biomolecule’s native ground conformational state in addition to its 3D structure. Characterizing biomolecules as holistic kinetic ensembles of ground and excited states can provide crucial insights into mechanistic understandings of their functions and further pave the way for new strategies in therapeutic development.
